# Blastische plasmazytoide dendritische Zellneoplasie (BPDCN): Eine seltene hämatologische Neoplasie mit häufiger kutaner Beteiligung

**DOI:** 10.1007/s00105-023-05192-9

**Published:** 2023-07-05

**Authors:** Katrin Nguyen, Sören Korsing, Yasmine Mansour, Katharina Meier

**Affiliations:** Klinik für Dermatologie, Allergologie und Venerologie, Charité – Universitätsmedizin Berlin, Charitéplatz 1, 10117 Berlin, Deutschland

**Keywords:** Blastisches NK-Zell-Lymphom, Basophile Blasten, CD123+, Zielgerichtete Therapie, Tagraxofusp, Blastic NK-cell lymphoma, Basophilic blasts, CD123+, Targeted therapy, Tagraxofusp

## Abstract

Die blastische plasmazytoide dendritische Zellneoplasie ist eine seltene hämatologische Neoplasie, die aus Vorläuferzellen der plasmazytoiden dendritischen Zellen entsteht und durch disseminierte, erythematöse bis bläulich-livide Plaques oder Nodi gekennzeichnet ist. Aufgrund der Seltenheit der Erkrankung stellen die Diagnose und Therapie eine Herausforderung dar. Wir berichten über einen Patienten mit BPDCN und stellen klinische und diagnostische Merkmale sowie Therapieoptionen vor.

## Abstract (en)

Blastic plasmacytoid dendritic cell neoplasm (BPDCN) is a rare hematological malignancy that derives from precursors of plasmacytoid dendritic cells and is characterized by disseminated, erythematous or bluish-livid plaques or nodi. Because of the disease’s rarity the diagnosis and treatment still pose a significant challenge. We present a case of a patient with BPDCN and show clinical and diagnostic characteristics as well as potential treatment regimes.

## Falldarstellung

### Anamnese

Ein 78-jähriger Patient stellte sich mit disseminierten Makulae und Plaques an Stamm und Extremitäten in unserer Klinik vor. Etwa 6 Monate zuvor sei initial eine solitäre Läsion an der rechten Brust aufgefallen. Im Verlauf entwickelten sich multiple Läsionen. Diese seien insbesondere am Rücken von einem moderaten Juckreiz (5/10 nach numerischer Analogskala (NAS)) begleitet. Weiterhin berichtete der Patient über eine allgemeine Abgeschlagenheit und Nachtschweiß. Subfebrile oder febrile Temperaturen seien nicht aufgefallen. Der Appetit sei unverändert, allerdings wurde ein Gewichtsverlust von 3 kg in ca. 6 Wochen festgestellt. An weiteren Erkrankungen waren eine arterielle Hypertonie, Hypercholesterinämie, Steatosis hepatis sowie ein Diabetes mellitus Typ 2 bekannt. Zudem wurde ein lokales Prostatakarzinom mittels Radiatio vor 3 Jahren behandelt. Der Patient nahm Nebivolol, Candesartan, Hydrochlorothiazid, Torasemid, Atorvastatin, Metformin und Tamsulosin ein.

### Klinischer Befund

**Figure 1:**
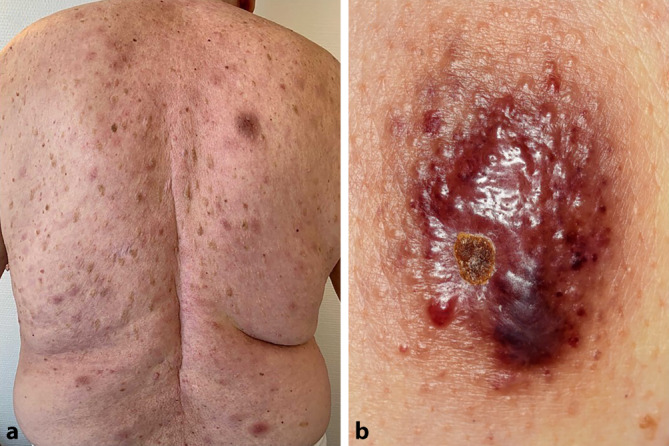

Bei der Inspektion fielen multiple, erythematöse bis bräunliche, teils livide anmutende, scharf und unscharf begrenzte, disseminierte Makulae und leicht infiltrierte Plaques variabler Größe am Stamm und an den Extremitäten auf. Am Rücken zeigte sich eine tannenbaumartige Anordnung. Teilweise imponierte ein kontusiformer Aspekt (Abb. 1a, b). Eine epitheliale Beteiligung ließ sich klinisch nicht eruieren. Palpatorisch fiel eine Lymphadenopathie zervikal beidseits und axillär rechts auf. Der weitere Haut- und Schleimhaut- sowie der internistische Untersuchungsstatus waren unauffällig.

### Weiterführende Diagnostik

Das Differenzialblutbild zeigte bis auf eine leichte Erythrozytopenie und eine normozytäre, normochrome Anämie keine Auffälligkeiten. Das Beta-2-Mikroglobulin war grenzwertig erhöht.

Die Entzündungsparameter, Elektrolyte, LDH, Leber- und Nierenwerte verblieben unauffällig.

**Figure 2:**
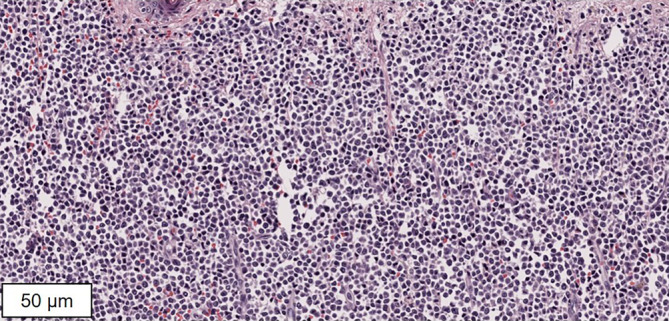

Es erfolgten multiple Hautbiopsien. In der histopathologischen Untersuchung stellte sich die Epidermis weitgehend unauffällig dar. Direkt unterhalb der Epidermis und bis in die tiefe Subkutis hineinreichend zeigten sich relativ monomorphe klein- bis mittelgroßzellige Infiltrate mit erhöhter mitotischer Aktivität (Abb. 2).

In der Lymphknotensonographie und in der Ganzkörpercomputertomographie stellten sich axillär rechts suspekte Lymphknoten dar, sodass eine Lymphknotenexstirpation erfolgte. Hier zeigten sich histopathologisch eine vollständig aufgehobene Gewebearchitektur und diffuse Infiltrate aus blastären Zellen mit Ausbreitung in die umgebende Subkutis. Die monomorphen Zellen des Infiltrats wiesen ein schmales Zytoplasma und einen hyperchromatischen Zellkern mit prominenten Nukleolen auf. Zudem fanden sich zahlreiche atypische Mitosefiguren sowie Apoptosekörperchen.

**Figure 3:**
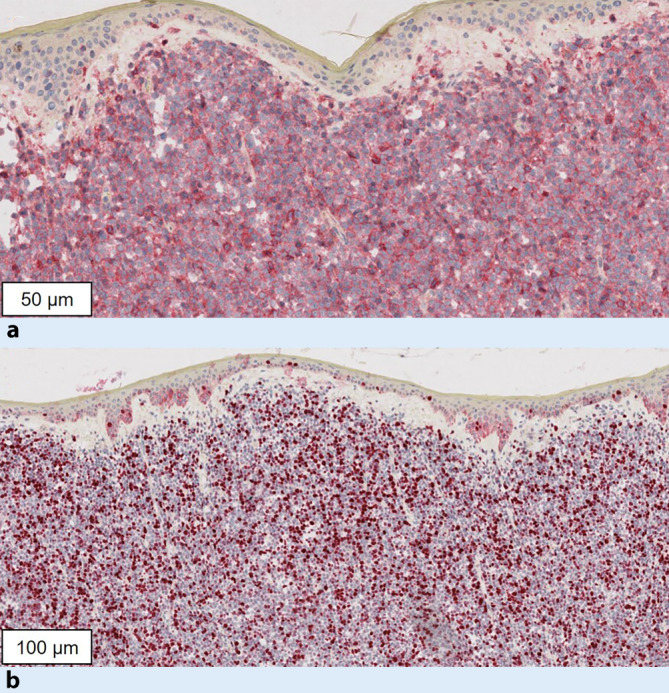

In der immunhistologischen Untersuchung fanden sich im Haut- und Lymphknotengewebe monomorphe CD4-, CD123- und TLC1-positive, stark proliferationsaktive Zellen (Ki-67: ca. 50 %) bei schwacher Reaktivität für CD56 (Abb. 3a, b). B‑ und T‑Zell-Marker waren negativ.

Eine Knochenmarkbiopsie, Abdomensonographie und Röntgenaufnahme des Thorax waren unauffällig.

### Diagnose und Verlauf

In Zusammenschau der Befunde stellten wir die Diagnose einer blastischen plasmazytoiden dendritischen Zellneoplasie (BPDCN). Es erfolgten die hämatoonkologische Anbindung sowie die Initiierung einer systemischen Therapie mit Tagraxofusp. Darunter zeigte sich ein Befundprogress mit Knochenmark- und Leberinfiltration, sodass nach 2 Therapiezyklen auf Azacitidin und Venetoclax und später auf Dexamethason und Cyclophosphamid sowie Vincristin und Dexamethason umgestellt wurde. Hierunter kam es bedauerlicherweise zu einer Sepsis, infolge derer der Patient verstarb.

## Diskussion

Die BPDCN ist eine seltene, aggressive, maligne Erkrankung mit ungünstiger Prognose. Sie macht etwa 0,44 % aller hämatologischen Neoplasien aus und tritt bevorzugt bei Männern (M:F, 3:1) in der 7. Lebensdekade auf. Die Erkrankung kann sich in jedem Lebensalter manifestieren, wobei sich 2 Häufigkeitsgipfel zeigen: > 60 und < 20 Jahre [8, 9].

Eine sich über viele Jahre häufig geänderte Nomenklatur umfasste Bezeichnungen wie „CD4^+^/CD56^+^ hämatodermale Neoplasie“ oder „blastisches NK-Zell-Lymphom“ bis die Erkrankung in der WHO-Klassifikation 2008 zur BPDCN umbenannt wurde. Seit 2016 stellt die BPDCN in der WHO-Klassifikation eine eigene Entität innerhalb der Gruppe der myeloischen Neoplasien und akuten Leukämien dar [1].

Die BPDCN entsteht durch klonale Proliferation von Vorläuferzellen der plasmazytoiden dendritischen Zellen. Klinisch manifestiert sich die BPDCN isoliert mit kutanen Läsionen oder mit einer zusätzlichen Beteiligung von Knochenmark, Lymphknoten oder anderen extramedullären Organen bis hin zum Befall des zentralen Nervensystems [6]. Häufig finden sich als Erstmanifestation solitäre oder multiple Hautläsionen von bis zu 10 cm Durchmesser und in Form von erythematösen bis bläulich-lividen, infiltrierten Plaques oder Nodi. Diese treten insbesondere an Stamm, Extremitäten und Gesicht auf, können aber am gesamten Integument lokalisiert sein [3]. In seltenen Fällen bleibt eine kutane Beteiligung aus, was die ohnehin schwierige Diagnosestellung erheblich erschwert [6]. Durch eine Beteiligung des Knochenmarks kann mitunter eine konsekutive Zytopenie resultieren, insbesondere eine Thrombozytopenie [2, 6]. Oftmals ist die BPDCN dadurch nicht einfach von einer akuten myeloischen Leukämie (AML) zu differenzieren [5, 8]. Durch die vielfältige Darstellung der BPDCN können differenzialdiagnostisch zudem sämtliche kutane Non-Hodgkin-Lymphome erwogen werden, wobei mittels Histopathologie und Immunhistochemie differenziert werden kann. Eine molekularbiologische Klonalitätsanalyse, eine Durchflusszytometrie sowie eine Knochenmarkdiagnostik geben weiteren Aufschluss über die zugrunde liegende Entität.

Bei der BPDCN finden sich klein- bis mittelgroße, diffus in der Dermis proliferierende, basophile Blasten mit häufig randständig gelegenem Zellkern sowie zahlreichen Mitosen [2, 5]. Immunphänotypisch zeigen sich die Zellen typischerweise positiv für CD123, CD4, CD56 und TCL‑1 sowie negativ für spezifische Marker anderer Zelllinien. Darüber hinaus können weitere Antigene wie HLA-DR, CD7, CD33 oder CD303 koexprimiert sein [10].

Die Erstlinientherapie der BPDCN ist der CD123-Antikörper Tagraxofusp (SL-401), ein Diphtherietoxin-Interleukin-13-Fusionsprotein. Es ist die erste für die BPDCN zugelassene Therapie, die sich gezielt gegen die CD123-exprimierenden Zellen richtet [7]. Weitere zielgerichtete Therapieansätze wie BET-Inhibitoren oder der BCL2-Inhibitor Venetoclax sind aktuell Gegenstand der Forschung [5]. Alternativ kommen Polychemotherapieregimes wie bei akuten Leukämien oder Lymphomen zum Einsatz [5]. Trotz initialen Ansprechens auf eine Chemotherapie kommt es häufig zu Rezidiven. Die mediane Überlebenszeit beträgt 12 bis 14 Monate [3]. Eine während der ersten Remission durchgeführte allogene Stammzelltransplantation (SZT) verlängert das mediane Überleben auf 23 Monate [6]. Eine Alternative bei fehlendem Donor für eine allogene SZT stellt die autologe SZT dar [4].

## Fazit für die Praxis

- Die BPDCN ist eine blastäre Neoplasie der plasmazytoiden dendritischen Zellen häufig mit Erstmanifestation in Form von kutanen, erythematösen bis bläulich-lividen, infiltrierten Plaques oder Nodi.
- Der Immunphänotyp der blastären Zellen ist durch Positivität für CD123, CD4, TLC‑1 und CD56 gekennzeichnet.
- Die Erstlinientherapie sollte als Monotherapie mit dem CD123-Antikörper Tagraxofusp erfolgen.
